# A potential link between *AQP3* and *SLC14A1* gene expression level and clinical parameters of maintenance hemodialysis patients

**DOI:** 10.1186/s12882-022-02922-4

**Published:** 2022-08-29

**Authors:** Rafał Zwiech, Agnieszka Bruzda-Zwiech, Ewa Balcerczak, Joanna Szczepańska, Adrian Krygier, Beata Małachowska, Dominika Michałek, Dagmara Szmajda-Krygier

**Affiliations:** 1 Dialysis Department, Norbert Barlicki Memorial Teaching Hospital, No. 1, Lodz, Poland; 2 Department of Pediatric Dentistry, Medical University of Lodz, Lodz, Poland; 3 Department of Pharmaceutical Biochemistry and Molecular Diagnostics, Medical University of Lodz, Lodz, Poland; 4 Department of Biostatistics and Translational Medicine, Medical University of Lodz, Lodz, Poland

**Keywords:** Aquaporin-3 (*AQP3*), End-stage kidney disease, Genes expression, Hemodialysis, UT-B (*SLC14A1*)

## Abstract

**Background:**

The transport of water and urea through the erythrocyte membrane is facilitated by aquaporins such as aquaglyceroporin (AQP3), and type B urea transporters (UT-B). As they may play an important role in osmotic balance of maintenance hemodialysis (HD) patients, the aim of the present study was to determine whether any relationship exists between the expression of their genes and the biochemical / clinical parameters in HD patients.

**Methods:**

*AQP3* and *UT-B* (*SLC14A1*) gene expression was evaluated using RT-qPCR analysis in 76 HD patients and 35 participants with no kidney failure.

**Results:**

The HD group demonstrated significantly higher median expression of *AQP3* and *UT-B* (Z = 2.16; *P* = 0.03 and Z = 8.82; *p* < 0.0001, respectively) than controls. *AQP3* negatively correlated with pre-dialysis urea serum concentration (*R =* -0.22; *P* = 0.049) and sodium gradient (*R =* -0.31; *P* = 0.04); however, no significant *UT-B* correlations were observed. Regarding the cause of end-stage kidney disease, *AQP3* expression positively correlated with erythropoietin dosages in the chronic glomerulonephritis (GN) subgroup (*R =* 0.6; *P* = 0.003), but negatively in the diabetic nephropathy subgroup (*R =* -0.59; *P* = 0.004). *UT-B* positively correlated with inter-dialytic weight gain% in the GN subgroup (*R =* 0.47; *P* = 0.03).

**Conclusion:**

Maintenance hemodialysis seems significantly modify *AQP3* and *UT-B* expression but their link to clinical and biochemical parameters needs further large-scale evaluation.

## Background

Chronic Kidney Disease (CKD) is accompanied by the retention of uremic toxins, which results in the development of uremic syndrome and its complications [1]. In 2016, 2,455,004 patients were treated for end-stage kidney disease (ESKD) in 79 countries reporting data to the United States Renal Data System [2]. Based on the International Society of Nephrology’s 2019 Global Kidney Health Atlas survey of 91 participating countries, the average number of people receiving Kidney Replacement Therapy (KRT) globally was 759 per million population (pmp) and the average number of new ESKD diagnoses worldwide was 144 individuals pmp [3]. Although kidney transplantation is preferred, hemodialysis (HD) remains the most common form of KRT [2, 3]. Unfortunately, patients with ESKD treated with in-center hemodialysis demonstrate poor overall survival, mainly due to high cardiovascular mortality resulting primarily from volume overload and hypertension [4]. In addition, side effects such as hypotension or disequilibrium syndrome can also result from the use of HD itself [5].

The effectiveness of HD is determined by the velocity of transluminal water and toxin transit. Although most cell membranes exhibit some degree of water permeability as a result of simple diffusion through the lipid bilayer, those of cells in certain organs express specific water channel proteins, known as aquaporins (AQP), that form aqueous pores across the plasma membrane [6]. The rate of transluminal shifts seems to primarily depend on the expression of AQP and the channels that play key roles in managing the changes in toxin concentrations within those three fluid spaces; if highly efficient, it can shorten the exposure of cells to the adverse effects of transmembrane pressure differences.

Thirteen types of AQP (0–12) are known to exist in humans [7]. Two are expressed on human erythrocytes (RBC): the water-conducting AQP1 and aquaglyceroporin AQP3. AQP3 uniquely allows water and glycerol and, to lesser extent, urea to pass through the membrane [8–12]. In human erythrocytes, AQP1 has a copy number of 59 000 and AQP3 of 2500; in addition, AQP3 appears to be about 2.4-times as water-permeable as AQP1, and has an affinity to glycerol of approximately 500/M [11]. The presence of AQP3 in RBCs could protect them from osmotic stress associated with exposure to high glycerol concentrations [12]. A similar hypothesis has been advanced by Macey and Yousef [13], who propose that such a rapid urea transport system protects RBCs from hypertonic damage in the renal medulla.

Another family of membrane proteins that selectively transport urea by creating a urea gradient across cell membranes are urea transporters (UTs). Two mammalian UT subfamilies have been discovered: UT-A, coded for by the gene *SLC14A2*, and UT-B, coded for by *SLC14A1*. The UT-B subfamily has two isoforms (UT-B1, UT-B2). In humans, the *SLC14A1* gene for UT-B1 is highly expressed in the plasma membrane of erythrocytes, and in other organs e.g. renal descending vasa recta, brain astrocyte, testis Sertoli cell, urothelial cell in bladder and ureter, and the endothelial cells in blood vessels [14–16]. In the plasma membrane of erythrocytes, UT-B has been identified as the Jk antigen, which determines the Kidd blood type in humans [16, 17].

UT-B is able to transport water in mouse erythrocytes, and it has been found to demonstrate very similar single-channel water permeability to AQP1 (7.5 × 10^–14^ cm^3^/s) [15]; however, due to the fact that fewer copies of UT-B (14 000 copies) are present than AQP1, the absolute contribution of UT-B to total water transport in normal erythrocytes is only about 8%.

Although the membrane expression of AQP1 in the RBCs of HD patients has been previously studied [8], little if anything is known about the expression of AQP3 and UT-B in this group of patients. Since maintenance hemodialysis patients are chronically exposed to the rapid water and toxins movements between water spaces, the equalizing transluminal osmotic pressure is crucial to reduce its damaging potential. These should enhance the channels efficiency or increase their numbers. Thus, we hypothesized that overexpression of genes encoding these channels may be noticeable in HD patients. In individuals in whom the expression of *AQP3* and *SLC14A1* would be higher than average, the better dialysis adequacy and improvement of other clinical parameters (e.g. hemoglobin, inter-dialysis weight gain, sodium gradients and erythropoietin dosing) might be expected. Therefore, the aim of the present study was to evaluate if any relationship exists between biochemical/ clinical parameters and the relative expression of *AQP3* and *UT-B* genes in patients receiving maintenance hemodialysis compared to controls.

## Methods

The study was conducted in compliance with the principles of the Helsinki Declaration. The study protocol was approved by the Medical University of Lodz Bioethics Committee, Resolution Number RNN 317/17/KE. According to principles of GCP (Good Clinical Practice), informed consent was obtained from all patients prior to their inclusion in the study.

### Recruitment of participants and data collection

The study included 76 patients receiving maintenance hemodialysis (49 men and 27 women), median age 65 years, including 25 patients with diabetes treated with insulin. All patients were recruited from the Dialysis Department of the Norbert Barlicki Memorial Teaching Hospital No. 1.

Kidney replacement therapy was performed exclusively with Fresenius 4008 HD machines, using standard bicarbonate dialysate fluid (comprising 140 mmol/L of sodium, 1.25 mmol/L of calcium and 0.75 mmol/L of magnesium). The potassium content varied depending on the level of kalemia prior to the HD session. The dialysis adequacy was evaluated using a single pooled *Kt / V* with an average value of 1.2–1.4 and the urea reduction ratio (URR). The dry weight among patients was assessed based on the results of a clinical examination, blood pressure (BP) measurement and whole body composition spectroscopy (BCM-Fresenius® Medical Care device) [18]. Mineral and bone disorders related to renal anemia and chronic kidney disease were effectively treated among all participants according to the KDOQI recommendations [19, 20], as was diabetes [21]. In the majority of patients, the antihypertensive treatment succeeded to maintain BP below 140/90 mmHg before and 130/80 mmHg after HD session.

The inclusion criteria for the study were as follows: age range 18–80 years, fixed frequency (thrice-weekly) HD regimen and clinically stable status. The exclusion criteria included uncontrolled hypertension or episodes of recurrent symptomatic hypotension, chronic NYHA class 4 heart failure and any cardiovascular event 3 months before recruitment to the study, severe acute infection requiring hospitalization.

The biochemical measurements were carried out routinely in certified central hospital laboratory automatic analyzers. All assessments, i.e. blood specimens and clinical evaluations, were conducted according to single time point assessment: one mid-week HD session.

At the time point, inter-dialytic weight gain (IWG), defined as the difference between current body mass and dry weight, was also measured as well as other clinical data. Pre-dialysis sodium gradient was also calculated, i.e. the difference between serum sodium level and dialysis fluid sodium level, presented as absolute numbers.

For the standardized variables, several ratios were recalculated as comparable ratios e.g. erythropoiesis stimulating agents (ESA) doses were counted per short-acting erythropoietin (EPO) and divided by dry body mass; inter-dialytic weight gain was transformed to IWG% and ultrafiltration (UF) ratio – ultrafiltration velocity given as IWG divided by dialyzer surface and session time.

A sex- and age-matched control group but with no kidney failure was also formed for the sake of comparison. This group included 35 participants.

### *AQP3* and *UT-B* (*SLC14A1*) expression analysis

The expression of the *AQP3* and *UT-B* (*SLC14A1*) genes was evaluated using reverse transcription-quantitative polymerase chain reaction (RT-qPCR) in the Department of Pharmaceutical Biochemistry and Molecular Diagnostics, Medical University of Lodz.

Briefly, total RNA was extracted from the peripheral blood cells of all patients and control group members using the Total RNA Mini kit (A&A Biotechnology, Gdynia, Poland) following the manufacturer's protocol. The A260/280 ratio (DNA/RNA absorbance to protein absorbance) was used to assess the purity of obtained RNA samples. The volume of RNA necessary for the reverse transcription reaction was calculated on the basis of absorbance at 260 nm. After isolation, RNA preparations were stored at − 76 °C pending analysis.

The obtained RNA samples were reverse-transcribed into cDNA using a High Capacity cDNA Reverse Transcription kit (Applied Biosystems; Thermo Fisher Scientific, Inc., Waltham, MA, USA), according to the manufacturer's protocol. The following parameters were used for the RT-PCR reaction: 25 °C for 10 min, then 37 °C for 120 min and 85 °C for 5 min. The final concentration of RNA in the reaction mixture in all samples was 0.005 µg/µl.

The presence of cDNA was verified by PCR amplification of the *GAPDH* (Glyceraldehyde 3-phosphate dehydrogenase) gene; performed using the AccuTaq™LA DNA Polymerase kit (Sigma Aldrich; Merck KGaA, Darmstadt, Germany), according to the manufacturer's protocol. The reaction mixture consisted of 1 µl cDNA template, 0.7 µL of 10 µM each primer, 3.5 µL of 1.5 mM 10 × PCR buffer without MgCl_2_ (Sigma Aldrich; Merck KGaA), 0.7 µL of 25 mM MgCl_2_ reagent, 0.4 µL of 0.2 mM dNTP (deoxynucleotide) mix, 0.2 µL of 0.5 U AccuTaq LA DNA Polymerase and distilled water to the final volume of 20 µL. A negative control containing distilled water instead of the cDNA template was included in every experiment. Amplification was performed using MJ Mini Personal Thermal Cycler (Bio-Rad Laboratories, Inc., Hercules, CA, USA). The thermocycling conditions for the *GAPDH* gene were as follows: Initial denaturation at 96 °C for 1 min, denaturation at 94 °C for 50 s, primer annealing at 58 °C for 50 s, elongation at 72 °C for 1 min and final elongation at 72 °C for 7 min.

The mRNA expression of the *AQP3* and *SLC14A1* genes was evaluated by PCR. Amplification was performed with the same procedure as for the *GAPDH* gene. The thermocycling conditions were as follows: Initial denaturation at 94 °C for 3 min, 34 cycles of denaturation at 94 °C for 50 s, primer annealing at 58 °C for 1 min for *AQP3* and 60 °C for 1 min for *SLC14A1* gene, elongation at 72 °C for 1 min and final elongation at 72 °C for 7 min. The PCR amplification products were separated by PAGE on a 2% gel. The size of the reaction products were 209 bp for *AQP3* and 231 bp for *SLC14A1*.

*AQP3* and *SLC14A1* mRNA expression level was assessed using qPCR performed in a Rotor-Gene™ 6000 thermocycler (Corbett Life Science; Qiagen GmbH, Hilden, Germany). The primer sequences were as follows: F 5’-GTGAAGCACAAGGAGCAGAT-3’, R: 5’-TAAGGTGCTATTTGGGCAAG-3’ for *AQP3* and F 5’- GAATGGACGGTCTTTGATTG-3’, R 5’- ACCTGGTTTTCACCCCTAAC-3’ for *SLC14A1*. The reaction mixture consisted of 5 Ll RT HS-PCR Mix Sybr® B (A&A Biotechnology, Gdynia, Poland), 0.7 µL of 10 µM each primer, 1 µL cDNA template and nuclease-free water to a final volume of 10 µL. The thermocycling conditions were as follows: Initial denaturation at 95 °C for 10 min, 40 cycles of denaturation at 95 °C for 10 s, primer annealing for 1 min (58 °C for *AQP3* and 60 °C for *SLC14A1*), elongation at 72 °C for 20 s.

Melting curve analysis was performed following amplification. The investigated and reference genes were amplified in triplicate, in separate tubes, during the same PCR run. A reference gene was used as an internal positive control and as a normalizer for the correction of gene expression. Relative changes in gene expression, determined by RTqPCR- analysis, were estimated based on the 2^–ΔΔCq^ method.

### Statistical analysis

Statistical analyzes were performed using STASTISTICA 13.1 (TIBCO Software, Palo Alto, CA). Nominal variables were presented as numbers with an appropriate percentage, and continuous variables were presented in the form of a median with the lower and upper quartiles (25–75 percentile). The normality of the distribution was tested by the Shapiro–Wilk W-test. The differences between the two groups were assessed using the Mann–Whitney U-test. The differences between more than two groups were assessed using the Kruskal–Wallis H-test. Correlations were calculated using the Spearman’s rank correlation test. In all conducted tests, a *P* value below 0.05 was assumed as significant. According to post hoc power test analysis for a study with 76 HD subjects and 35 control subjects we were able to detect a true difference in the gene expression of [0.578] of SD with power of 80%. The Type I error probability was associated with the test as 0.05.

## Results

In the study group, the median time for starting HD was 28 months, with the minimum time being six months. The median HD session time was 240 min. The causes of end-stage kidney disease included chronic glomerulonephritis (GN) in 22 patients, diabetes mellitus (DM) in 22, hypertension (AH) in 16 and other (viz. polycystic kidney disease, vasculitis, chronic tubulo-interstitial nephritis, Alport’s syndrome and unidentified) in 16 patients. The characteristics of the study group and clinical data are presented in Tables 1 and 2.

**Table 1:** Study group characteristics

	***N* = 76 (%)**
**Sex**	
Males	49 (64.47%)
Females	27 (35.53%)
**Cause of end-stage kidney disease**	
Glomerulonephritis	22 (28.95%)
Arterial hypertension	16 (21.05%)
Diabetes mellitus	22 (28.95%)
Other	16 (21.05%)

**Table 2:** Study group characteristic and clinical/biochemical data

Characteristic	Me (25–75%)
Age, years	65.00 (55.50 – 72.00)
Hemodialysis vintage, months	28.00 (22.00 – 39.00)
Urea before hemodialysis, mg/dL	124.00 (103.50 – 150.50)
Urea after hemodialysis, mg/dL	37.00 (30.00 – 45.00)
Urea reduction ratio (URR), %	70.40 (66.52 – 74.01)
Hgb, g/dL	10.85 (10.00 – 11.80)
EPO ratio, IU/kg of dry body mass	86.83 (30.09 – 134.47)
Interdialytic weight gain (IWG), %	3.43 (2.02 – 5.15)
UF ratio, kg/dialyser m^2^/h	9.01 (7.98 – 9.93)
Pre-dialysis sodium, mmol/L	138 (13 6 – 140)
Pre-dialysis sodium gradient	2.0 (1.0 – 3.0)
Hemodialysis session time, min	240.00 (235.00 – 270.00)
Residual renal function, mL/24 h	0.00 (0.00 – 200.00)

*Abbreviations*: *EPO* Erythropoietin, *Hgb* Hemoglobin, *UF* Ultrafiltration

The medians of expression values in the study group were 1.13 for *AQP3* and 16.57 for *UT-B* (*SLC14A1*). No differences in gene expression were observed between men and women, between diabetic and non-diabetic patients, or between those treated and not treated with ACEi / ARB. Despite primary disease, neither *AQP3* nor *UT-B* expression varied significantly with regard to cause of end-stage kidney disease (Table 3). In HD group, no significant correlations were found between most of the analyzed clinical data and the expression of the aquaporin or urea channel genes; however, significant negative correlations were observed between *AQP3* and pre-dialysis urea serum concentration (*R =*-0.22; *P* = 0.049) and pre-dialysis sodium gradient (*R =* -0.31; *P* = 0.04). More detailed results are shown in Table 4.

**Table 3:** *AQP3* and *UT-B (SLC14A1*) gene expression in study population with major coefficients comparison subdivisions

	***AQP3***		***UT-B (SLC14A1)***	
	**Me (25–75%)**	***P* value**	**Me (25–75%)**	***P* value**
All patients	1.13 (0.73 – 1.49)	n/a	16.57 (15.34 – 17.18)	n/a
Sex	M: 1.13 (0.60 – 1.46)	0.45	M: 16.92 (15.60 – 17.16)	0.63
	F: 1.09 (0.77 – 1.53)		F: 16.17 (15.15 – 17.45)	
DM	Y: 1.39 (0.92 – 1.82)	0.13	Y: 16.95 (15.51 – 17.16)	0.58
	N: 1.11 (0.57 – 1.43)		N: 16.17 (15.27 – 17.19)	
Treatment with ACEi or ARB	Y: 1.05 (0.76 – 1.43)	0.17	Y: 16.20 (15.15 – 17.11)	0.43
	N: 1.21 (0.68 – 1.82)		N: 16.92 (15.70 – 17.45)	
Residual kidney function	Y: 1.38 (0.64 – 1.68)	0.28	Y: 16.95 (16.09 – 17.47)	0.16
	N: 1.10 (0.76 – 1.44)		N: 16.06 (15.15 – 17.14)	
Cause of end-stage kidney disease	GN: 1.24 (0.88 – 1.50)	0.14	GN: 16.92 (15.92 – 17.19)	0.57
	AH: 1.07 (0.62 – 1.33)		AH: 15.88 (15.21 – 17.01)	
	DM: 1.30 (0.92 – 1.82)		DM: 16.76 (15.15 – 17.00)	
	Other: 0.84 (0.50 – 1.29)		Other: 16.99 (14.76 – 17.49)	

*Abbreviations*: *Me* Median, *n/a* Non applicable, *ACEi or ARB* Angiotensin converting enzyme and receptor blockers, *AH* Arterial hypertension, *DM* Diabetes mellitus, *F* Females, *GN* Glomerulonephritis, *M* Males, *N* No, *Y* Yes

**Table 4:** Correlations between *AQP3, UT-B (SLC14A1)* gene expression and clinical data in the study group

	***AQP3***		***UT-B (SLC14A1)***	
	**R**	***P* value**	**R**	***P* value**
Age, years	0.10	0.39	0.01	0.95
Sodium gradient (pre-dialysis)	**-0.31**	**0.04**	0.19	0.77
Urea before hemodialysis, mg/dL	**-0.22**	**0.049**	0.08	0.47
Urea after hemodialysis, mg/dL	-0.10	0.39	0.04	0.71
Urea reduction ratio (URR), %	-0.15	0.20	0.11	0.35
Hb, g/dL	-0.06	0.60	0.15	0.20
EPO ratio, IU/kg of dry body mass	0.03	0.83	-0.03	0.79
Interdialytic weight gain IWG, %	-0.12	0.31	0.12	0.30
UF ratio—IWG/dialyser, kg/dialyser m^2^/h	0.15	0.19	-0.10	0.37
Hemodialysis session time, min	-0.10	0.37	0.06	0.62
Hemodialysis vintage, months	-0.04	0.76	-0.13	0.26
Residual kidney function, mL/24 h	0.15	0.20	0.14	0.24

*Abbreviations*: *EPO* Erythropoietin, *Hgb* Hemoglobin, *UF* Ultrafiltration

The relationships between clinical data and the expression of *AQP3* and *UT-B (SLC14A1)* according to cause of end-stage kidney disease are given in Tables 5 and 6. *AQP3* negatively correlated with pre-dialysis urea and HD session time in subgroup *Other* ( *R =* -0.5; *P* = 0.046 and *R =* -0.61; *P* = 0.01, respectively). *AQP3* expression positively correlated with the EPO dosages in the GN subgroup (*R =* 0.6; *P* = 0.003) and negatively correlated with EPO doses in the DM subgroup (*R =* -0.59; *P* = 0.004).

**Table 5:** Correlations between *AQP3* gene expression and clinical data with subdivision of end-stage kidney disease causes

	**GN**		**AH**		**DM**		**Other**	
	**R**	***P* value**	**R**	***P* value**	**R**	***P* value**	**R**	***P* value**
Age, years	0.04	0.88	0.40	0.12	0.26	0.25	0.29	0.28
Urea before hemodialysis, mg/dL	-0.39	0.07	-0.15	0.58	0.02	0.92	**-0.50**	**0.046**
Urea after hemodialysis, mg/dL	-0.25	0.26	-0.20	0.46	0.09	0.68	-0.31	0.24
Urea reduction ratio (URR), %	-0.13	0.57	0.13	0.64	-0.15	0.50	-0.04	0.90
Hb, g/dL	-0.25	0.25	0.10	0.72	0.32	0.15	-0.44	0.09
EPO ratio, IU/kg of dry body mass	**0.60**	**0.003**	0.16	0.54	**-0.59**	**0.004**	0.15	0.57
Interdialytic weight gain IWG, %	-0.23	0.29	-0.19	0.49	0.07	0.77	0.04	0.87
Hemodialysis session time, min	0.04	0.85	-0.48	0.06	0.06	0.78	**-0.61**	**0.01**
UF ratio—IWG/dialyser, kg/dialyser m^2^/h	0.24	0.29	-0.28	0.29	0.28	0.20	-0.11	0.68
Hemodialysis vintage, months	0.11	0.64	-0.10	0.70	-0.24	0.28	0.06	0.82
Residual renal function, mL/24 h	0.01	0.96	0.25	0.35	0.36	0.10	-0.06	0.84

*Abbreviations*: *DM* Diabetes mellitus, *EPO* Erythropoietin, *GN* Primary glomerulonephritis, *AH* Arterial hypertension, *IWG* Inter-dialytic weight gain, *UF* Ultrafiltration

**Table 6:** Correlations between *UT-B* (*SLC14A1*) gene expression and clinical data with subdivision according to end-stage kidney disease causes

	**GN**		**AH**		**DM**		**Other**	
	**R**	***P* value**	**R**	***P* value**	**R**	***P* value**	**R**	***P* value**
Age, years	-0.21	0.34	0.48	0.06	0.02	0.93	0.14	0.62
Urea before hemodialysis, mg/dL	0.30	0.18	-0.43	0.09	0.21	0.35	0.05	0.86
Urea after hemodialysis, mg/dL	0.11	0.64	-0.20	0.46	0.16	0.47	-0.10	0.72
Urea reduction ratio (URR), %	0.23	0.30	-0.26	0.34	0.07	0.77	0.33	0.21
Hb, g/dL	0.17	0.45	-0.34	0.19	0.10	0.65	0.08	0.78
EPO ratio, IU/kg of dry body mass	-0.12	0.61	-0.30	0.26	-0.13	0.56	0.36	0.16
IWG, %	**0.47**	**0.03**	-0.28	0.29	0.40	0.06	-0.10	0.71
Hemodialysis session time, min	-0.13	0.57	0.37	0.16	0.23	0.31	0.01	0.96
UF ratio—IWG/dialyser, kg/dialyser m^2^/h	**-0.48**	**0.02**	**0.64**	**0.008**	0.08	0.72	-0.18	0.49
Hemodialysis vintage, months	< 0.01	0.99	**-0.66**	**0.005**	-0.19	0.40	0.23	0.39
Residual kidney function, mL/24 h	0.03	0.89	0.14	0.60	-0.07	0.76	0.39	0.14

*Abbreviations*: *DM* Diabetes mellitus, *EPO* Erythropoietin, *GN* Primary glomerulonephritis, *AH* Arterial hypertension, *IWG* Interdialytic weight gain, *UF* Ultrafiltration

In participants with glomerulonephritis as the cause of end-stage kidney disease, *UT-B* positively correlated with IWG% (*R =* 0.47; *P* = 0.03) and UF ratio negatively correlated with urea channel expression (*R =* -0.48; *P* = 0.02). In patients in whom arterial hypertension was a primary cause of kidney failure and HD, UF ratio positively correlated with *UT-B* (*R =* 0.64; *P* = 0.008). In the arterial hypertension group, *UT-B* expression negatively correlated with hemodialysis vintage (*R =* -0.66; *P* = 0.005); interestingly, this was the only group where this relationship was observed.

**Fig. 1:**
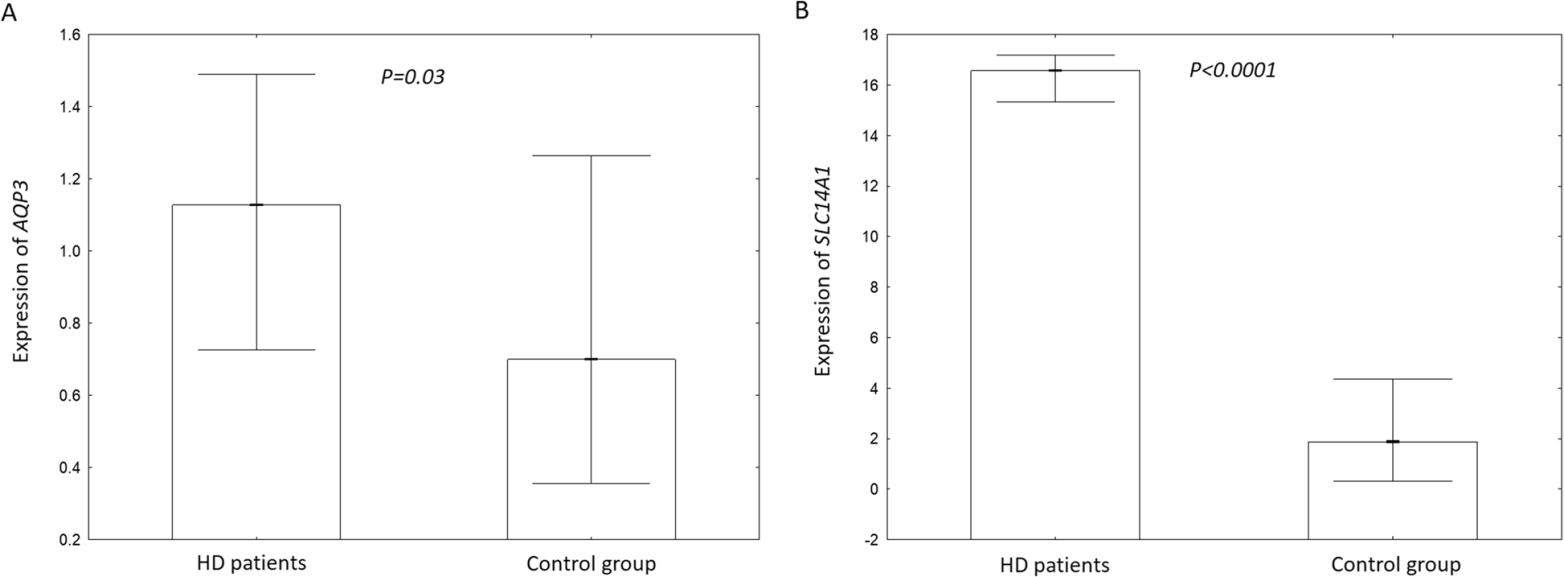
The mRNA expression of the *AQP3* (**A**) and *SLC14A1* (**B**) genes in hemodialysis patients and the control group

In the control group median *AQP3* was 0.70 (0.36 – 1.27) and median *UT-B* was 1.88 (0.31–4.36). These values were significantly lower than in the study (HD) group (Z = 2.16; *P* = 0.03 and Z = 8.82; *p* < 0.0001, respectively) (Fig. 1).

## Discussion

AQP3 facilitates the transluminal transport of water, glycerol and hydrogen peroxide [22]. Renal medullary aquaporin level has been found to be dysregulated in kidney diseases. For example, reduced *AQP3* mRNA expression has been associated with nephron loss and the development of chronic interstitial fibrosis [23]. In addition, a 70% decrease in AQP3 expression has been reported in the inner medulla, during induced nephrotic syndrome, which could be a physiological response to extracellular water reabsorption [24, 25]. Furthermore, AQP3 deletion in collecting ducts significantly impairs kidney function and markedly increases kidney damage, as well as renal oxidative stress, inflammation and apoptosis after renal ischemia–reperfusion (IR) [26]. It is believed that AQP3 exerts its protective effect on the collecting ducts during the process of renal IR by facilitating plasma membrane glycerol transport, and supporting energy metabolism, i.e. ATP synthesis and mitogen-activated protein kinase (MAPK) pathways.

The role of AQP3 has been well studied in kidney diseases that may impair kidney function, generally little is known of its activities in maintenance hemodialysis patients and its influence on the spectrum of clinical symptoms; however, epidermal AQP3 has been proven to be elevated in patients with chronic kidney disease-associated pruritus (CKD-aP) [27]. Our present findings indicate increased *AQP3* gene expression in the blood of HD patients compared to controls.

Although uremia, glucose and insulin have been found to affect *AQP3* gene expression, most studies have been performed in vitro [28, 29]. In vivo studies have rarely been performed and these have never correlated their findings with clinical data in HD patients as in the present study. In the present study, the HD patients demonstrated nearly twice the *AQP3* and *UT-B* expression level than healthy participants. Although these preliminary findings might be of interest, the possibility to discuss the overexpression of these genes in the HD group compared to the control group is limited, as our study is, to the best of our knowledge, the first on this subject. Presumably, this may be explained by the need for rapid fluid and urea movement to ameliorate and equalize transluminal osmotic pressure [30].

Physiologically, UT-B proteins function to protect erythrocytes from repeated osmotic pressure-related injury, thus maintaining their permeation and morphology. Rapid urea transport mediated by UT-B in erythrocyte may help establish urea concentration gradient [31]. An UT-B deficiency decreases the urea permeability of erythrocytes by 45 times [32].

Interestingly, the uremic milieu influences the kinetics of water and urea movements between blood and brain, reducing UT-B1 expression by 50% and increases AQP expression (AQP4, APQ 9) by 50% or more [5]. The authors suggest that during rapid removal of extracellular urea through fast dialysis, the low abundance of UT-B slows the escape of urea from astrocytes. The resulting osmotic gradient promotes water retention or entry into brain cells and subsequent brain swelling, due to the high level of AQPs. This molecular mechanism may well account for the occurrence of dialysis disequilibrium syndrome as a neurological complication of HD [5].

The vast majority of our obtained clinical data did not correlate with *AQP3* or *UT-B* gene expression. However, *AQP3* was found to be negatively associated with pre-dialysis urea serum concentration. Chronically uremic patients experience a steady state of hyperosmolality, in which urea equilibrates between the blood, tissues and extracellular extravessel space generating no osmotic gradient and thus no water movement [5]. Hemodialysis provokes rapid removal of urea from the blood; this results in the generation of an increased osmotic gradient that favors water movement into the cells. The reduced expression of *AQP3* in HD patients with higher pre-dialysis urea might be therefore regarded as a defense mechanism protecting cells from edema, making HD safer.

In human peritoneal mesothelial cells, exposure to osmotic agents such as glucose and mannitol, as in peritoneal dialysis, can uprate AQP3 protein synthesis and gene expression, leading to rapid transmembrane water transport [28]. It is possible that AQP3 may be involved in regulating the blood glucose level: AQP3 expression in Caco-2 cells was found to be suppressed by insulin [29]. Surprisingly, our current findings indicate that AQP3 synthesis was still higher in patients with coexisting DM following residual diuresis or ACEi /ARB treatment than those in healthy controls; however, these values were comparable to those in other groups of HD patients. This may suggest that expression of *AQP3* in HD patients may be upregulated not only by osmotic stimulation by glucose but also upon exposure to other factors. The mechanism of gene and protein AQP3 up-regulation after osmotic stimulation remains unclear; however, it is known to be upregulated by dehydration [28].

It is also possible that regardless of the cause of end-stage kidney disease, *AQP3* expression may be elevated to maintain cardiovascular stability in response to ultrafiltration during HD, i.e. by quickly shifting water from the intracellular to extracellular space. AQP3 plays a role in maintaining intracellular osmolality and cell volume by providing water to water-deprived cells, and it has also been suggested that AQP3 expression in the kidney and extrarenal organs occurs as a response to shifting tonicity. Hypertonic induction of AQP expression has been demonstrated in MDCK cells, and cells exposed to hypertonic medium containing either trisaccharide raffinose or NaCl have demonstrated similar changes in *AQP3* mRNA level [6]. In addition, hyperosmolarity increased *AQP3* mRNA expression by more than twofold in human keratinocyte cultures [33]. Exposure to hyperosmolar NaCl solutions also increased AQP3 protein expression in human urothelial cells [34]. The authors speculate that AQP3 responds to urine hypertonicity and mediates the diffusion of water and urea across the urothelium [34].

In the present study, *AQP3* expression negatively correlated with the pre-dialysis sodium gradient, calculated as the difference between serum and dialysis fluid sodium concentrations (140 mmol/L): lower pre-dialysis sodium gradients, due to higher serum sodium levels, were associated with higher *AQP3* levels. This may play a crucial role in ameliorating rapid shifts of “pure water” between intra and extracellular water spaces, especially when patients undergo HD. However, no significant difference in *AQP3* gene expression was found between DM subjects and other groups of HD patients; this observation may be attributed to the interactions of the osmotic stimulators: water translocation from the intracellular to the extracellular fluid driven by elevated serum glucose level [35] results in a relative decrease in serum sodium concentration, i.e. translocational hyponatremia [36].

Increased pre-dialysis sodium gradients has been found to be a potential contributory factor for increased IWG in HD patients [37]. Furthermore, some studies suggest that the reduction of sodium in the dialysate from 140 to 138 or 137 mmol/L, thus lowering its pre-dialysis gradient, seems to reduce inter-dialytic weight gain [38–40]. As such, based on these previous findings and the negative correlation observed between *AQP*3 expression and pre-dialysis sodium gradient in present study, the impact on *AQP3* expression should be obvious; however, no direct correlation was observed between IGW and *AQP3* expression.

Increased inter-dialytic weight gain is associated with erythropoietin resistance in maintenance hemodialysis patients [41]. In addition, the presence of extracellular fluid overloading in end-stage kidney disease HD patients has been associated with EPO hyporesponsiveness [42]. In our present study, it was found that patients with ESKD caused by glomerulonephritis, and with higher IWG, needed higher ESA doses to correct anemia. In addition, a strong negative correlation was found between *AQP3* and EPO ratio in patients with end-stage kidney disease caused by DM; median *AQP3* expression was also found to be higher in DM patients than non-DM patients (1.39 vs. 1.11), but this difference was not significant.

Glucose has been found to enhance the expression of AQP3, while insulin depletes it [28]. Higher absolute values of *AQP3* expression have been confirmed in DM subjects; this can ameliorate water transport both into and out of cells, resulting in water migration between intravascular space to extravascular intra- and extracellular spaces. Thus, despite the inter-dialytic weight gain observed in the DM population of HD patients, reductions in the volume of water may still occur in the blood vessels [43]. This could lead to elevated hemoglobin and hematocrit values in standard measurements and account for the phenomenon of reduced EPO dosage in DM patients.

In patients among whom ESKD was caused by glomerulonephritis, the expression of *UT-B* positively correlated with IWG%. This subgroup is often characterized by a younger age and higher urea production due to a higher protein diet, what prompts to use dialyzers of larger surfaces. This might explain why UF ratio negatively correlated with *UT-B* expression. In contrast, in the arterial hypertension subjects, who were usually older, less active and on a low-protein diet, *UT-B* positively correlated with ultrafiltration ratio, probably due to lower IWG%. These reasons might also account for the negative correlation observed between *UT-B* and hemodialysis vintage.

The main findings of this study were significantly higher expression levels of both, *AQP3* and *SLC14A1*, genes in HD patients compared to healthy participants, what seem to confirm the preliminary hypothesis, although the improvement in clinical characteristic was not observed. Nevertheless, this study has taken a first step in understanding of the role of UTB and AQP3 as water and urea transporters in end-stage kidney disease, yet there still remains much to be determined.

Our study has some limitations. The presented results are limited to the Polish population with a restricted sample size. This caused the subgroups to be small and might have resulted in a lack of association between certain demographic and clinical characteristics. In addition, as the expression of *AQP3* and *SLC14A1* genes in end-stage kidney disease patients has not been previously examined, our findings should be treated as preliminary and interpreted with care. Further studies are needed with larger numbers of subjects. Nevertheless, the process of chronic HD seem to modify the gene expression level of *AQP3* and *SLC14A1*, although the exact mechanism is still unknown. This may serve as the starting point for further analysis, which can be the base of more individualized hemodialysis therapy.

## Conclusions

Our findings indicate that maintenance hemodialysis may significantly modify the expression of *UT-B* and *AQP3*; although their link to clinical parameters remains unclear. Nevertheless, exposure to rapid water and the subsequent tonicity shifts taking place during a HD session may play an important role. However, our data regarding *UT-B* and *AQP3* level are promising, and sodium remains an important consideration when designing more individualized HD.

## Data Availability

All relevant data are included in the article. The datasets used and/or analysed during the current study are available from the corresponding author on reasonable request, due to sensitive data protection.

## Abbreviations

AQP3: Aquaglyceroporin 3
AH: Arterial hypertension
ACEi or ARB: Angiotensin converting enzyme and receptor blockers
CKD: Chronic Kidney Disease
DM: Diabetes mellitus
ESKD: End-stage kidney disease
HD: Hemodialysis
EPO: Erythropoietin
GN: Primary glomerulonephritis
Hgb: Hemoglobin
KRT: Kidney replacement therapy
RT-qPCR: Reverse transcription-quantitative polymerase chain reaction
*SLC14A1*: Gene for urea transporter B1 (UT-B1)
UF: Ultrafiltration
URR: Urea reduction ratio
UTs: Urea transporter-proteins that selectively transport urea
